# Companion cropping with potato onion enhances the disease resistance of tomato against *Verticillium dahliae*

**DOI:** 10.3389/fpls.2015.00726

**Published:** 2015-09-11

**Authors:** Xuepeng Fu, Xia Wu, Xingang Zhou, Shouwei Liu, Yanhui Shen, Fengzhi Wu

**Affiliations:** ^1^Department of Horticulture, Northeast Agricultural UniversityHarbin, China; ^2^Department of Life Science and Agroforestry, Qiqihar UniversityQiqihar, China; ^3^Department of Horticulture, Heilongjiang Bayi Agricultural UniversityDaqing, China

**Keywords:** companion cropping, disease resistance, interspecific interaction, RNA-seq, root exudates, soil-borne diseases, *Verticillium dahliae*

## Abstract

Intercropping could alleviate soil-borne diseases, however, few studies focused on the immunity of the host plant induced by the interspecific interactions. To test whether or not intercropping could enhance the disease resistance of host plant, we investigated the effect of companion cropping with potato onion on tomato Verticillium wilt caused by *Verticillium dahliae* (*V. dahliae*). To investigate the mechanisms, the root exudates were collected from tomato and potato onion which were grown together or separately, and were used to examine the antifungal activities against *V. dahliae in vitro*, respectively. Furthermore, RNA-seq was used to examine the expression pattern of genes related to disease resistance in tomato companied with potato onion compared to that in tomato grown alone, under the condition of infection with *V. dahliae*. The results showed that companion cropping with potato onion could alleviate the incidence and severity of tomato Verticillium wilt. The further studies revealed that the root exudates from tomato companied with potato onion significantly inhibited the mycelia growth and spore germination of *V. dahliae*. However, there were no significant effects on these two measurements for the root exudates from potato onion grown alone or from potato onion grown with tomato. RNA-seq data analysis showed the disease defense genes associated with pathogenesis-related proteins, biosynthesis of lignin, hormone metabolism and signal transduction were expressed much higher in the tomato companied with potato onion than those in the tomato grown alone, which indicated that these defense genes play important roles in tomato against *V. dahliae* infection, and meant that the disease resistance of tomato against *V. dahliae* was enhanced in the companion copping with potato onion. We proposed that companion cropping with potato onion could enhance the disease resistance of tomato against *V. dahliae* by regulating the expression of genes related to disease resistance response. This may be a potential mechanism for the management of soil-borne plant diseases in the intercropping system.

## Introduction

In modern agriculture intercropping has long been used for the increase of crop productivity and management of soil-borne diseases (Boudreau, 2013; Li et al., 2014). It was widely observed that intercropping was beneficial for the management of soil-borne disease, such as inhibition of pepper Phytophthora blight in maize/pepper intercropping system (Yang et al., 2014), suppression of watermelon Fusarium wilt in rice/watermelon intercropping (Ren et al., 2008), and inhibition of soybean red crown rot in maize/soybean intercropping (Gao et al., 2014) and so on.

There are two ways, by which intercropping can suppress soil-borne disease, one of them is to decrease the attacks by pathogens, and the other one is to increase disease resistance of host plant (Ratnadass et al., 2012; Boudreau, 2013). To date, most studies focused on the less pathogen attacks, by the alteration of microenvironment and formation of “root wall” to restrict the spread of spores (Gómez-Rodrıguez et al., 2003; Gao et al., 2014), or by the increase of soil microbial antagonism for pathogens (Ren et al., 2008; Fengzhi and Xingang, 2009), or/and by the direct inhibition of pathogens with root exudates from companied plants (Ren et al., 2008; Hao et al., 2010; Gao et al., 2014; Yang et al., 2014). In previous studies, the main focus was on the effects of root exudates from companied plant on the pathogen which attacks the host plant (Ren et al., 2008; Hao et al., 2010; Gao et al., 2014; Yang et al., 2014). However, whether or not the root exudates from host plant have antifungal activity on pathogen in intercropping was unknown.

In variable environments, plants have evolved defense systems to response to pathogen attacks (Chisholm et al., 2006). The potential agents in disease resistance system include pathogenesis-related proteins (PRs), defense enzymes, plant hormones and other defense related proteins (Shamrai, 2014). There were a few reports mentioned that intercropping increased the expression of PR genes, and enzymatic activity related to the defense of host plants in intercropping compared to monoculture (Gao et al., 2014; Xu et al., 2015a). However, Up to now, there was very limited knowledge regarding to the induction of disease resistance in host plant intercropped with other plant species. Whether or not the disease resistance of host plant was one of the mechanisms for the management of soil-borne plant diseases in the intercropping system was unknown.

Tomato (*Solanum lycopersicum*) is an important vegetable crop and cultivated worldwide. Unfortunately, tomato is a favorable host for *V. dahliae*, a soil-borne pathogen resulting in Verticillium wilt in many plant species (Klosterman et al., 2009). Potato onion (*Allium cepa* var. *agrogatum Don.)*, a variant of onion, is widely cultivated in Northeast of China, and was regard as a better companion plant in terms of disease and pests management in intercropping system according to farmer's practice. In this case, it is interesting to know whether potato onion as companion plant is efficient for controlling tomato Verticillium wilt. Based on the previous studies we hypothesized (i) companion cropping with potato onion could alleviate tomato Verticillium wilt, (ii) the root exudates from potato onion and tomato have antifungal activities on *V. dahliae* growth, (iii) companion cropping with potato onion could enhance the expression of defense genes related to disease resistance against *V. dahliae*. To confirm these, we determined the effect of potato onion as a companion crop on tomato Verticillium wilt, investigated the antifungal activities of the root exudates from potato onion and tomato on *V. dahliae* growth, and investigated the expression profile of the disease resistance related genes of tomato infected with *V. dahliae* by RNA-Seq. Finally, we explored the relationship among the incidence, the effect of root exudates and gene expression on disease resistance of tomato against *V. dahliae*.

## Methods and materials

### Cultivation of plants

The tomato seeds, Qiyanaifen (susceptible to *V. dahliae*), were purchased from Qiqihar Vegetable Research Institute (Qiqihar, China). Potato onion variety Suihua, a native variety with potential allelopathy (Liu et al., 2013), was provided by Laboratory of Vegetables Physiological Ecology (harbin, China). Tomato seeds were surface sterilized with 3.8% sodium hypochlorite for 10 min and afterwards rinsed three times with sterile distilled water. Then germinated in a mixture of peat: perlite (1:1 v/v). Before transplanting, the tomato seedlings were cultivated in the greenhouse located in the Experimental Center of the Northeast Agricultural University, Harbin, China (45°41′N, 126°37′E). Potato onion was stored at 4°C before planting.

### Fungal culture

The *V. dahliae* race 1 (Vd1) was provided by Tomato Breeding Center of Northeast Agricultural University (harbin, China). Spore suspensions were prepared and gave a final concentration of 1.0 × 10^7^ spores·mL^−1^ sterile 0.5% gelatin solution (Pegg and Street, 1984; Dobinson et al., 1996). The pathogenicity of Vd1 was tested by dipping the tomato roots into a spore suspension (1.0 × 10^7^ mL^−1^) and replanted in autoclaved soil in our preliminary trial (Orenstein et al., 1989). After the plants were treated for 15 days, the symptoms of Verticillium wilt were observed.

### Determination of the incidence and symptom scoring of tomato verticillium wilt

The pot culture experiments were conducted in the greenhouse to test the effect of companion cropping with potato onion on tomato Verticillium wilt in 2013 and 2014, respectively. The tomato seedlings with four true—leaf were transplanted into pots (17 cm diam, 19 cm height) filled with autoclaved field soils according to the methods described by van Wees et al. (2000). The soils were autoclaved twice for 20 min with a 24 h interval, thus the microbes in the soils were eliminated (examined by plate cultivation method, data unshown). The pot experiments were conducted with three blocks, each containing two cropping treatments. One was tomato/potato onion companion cropping, which meant one tomato seedling grew with two potato onion bulbs with the distance of 10 cm between tomato and potato onion bulbs, as TC treatment (TC). The other one was tomato monoculture, which meant one tomato seedling grew alone (without potato onion) in the pot, served as TM treatment (TM). Each treatment contained 10 pots (10 TC or 10 TM). The arrangement of the pots was randomized completely. Before planted into the pot, the roots of tomato seedlings and potato onion bulbs were cleaned with tap water first, then washed with autoclaved water for three times. Autoclaved water was applied throughout the experiment. The weeds were removed manually.

Twenty days after transplanting, the seedlings of both treatments were inoculated with 20 mL spore suspension (1 × 10^7^ spores·mL^−1^) solution of Vd1 by pouring into rhizosphere of each tomato seedling (Gao et al., 2014). The incidence and the symptoms scoring of Verticillium wilt were observed and calculated from 10 to 30 days after the inoculation (DAI), with a 3 days interval. The incidence was defined as the percentage of tomato seedlings with disease symptoms in all treated seedlings in each treatment. The symptoms scoring was evaluated by 0–5 scale based on the numbers of leaves with disease symptoms (Shittu et al., 2009), that is: 0, healthy; 0.5, premature loss of both cotyledons; 1.0, yellowing and flaccidity of the first leaf; 2.0, lower 40% of leaves infected; 3, lower 60% of leaves infected; 4, lower 80% of leaves infected; 5, plant dead. Stunting (>2.5 cm shorter than control) contributes an additional 0.5 to the disease score of each plant. The incidence and the symptoms scoring were evaluated independently by two observers and averaged.

### Test of antifungal activities of root exudates *in vitro*

The tomato seedlings with four true leaves were cultivated in a plant growth chamber at 25°C with a photoperiod of 16 h light/8 h dark. There were four treatments in this study, which were TC and TM (same as greenhouse studies), OC (potato onion grown with tomato) and OM (potato onion grown alone). The inoculation was the same as greenhouse studies mentioned above at 20th after companied with potato onion. The collection of root exudates was carried out according to the method reported previously (Ren et al., 2008; Li et al., 2013) at 7 DAI with a little modification. Briefly, roots of tomato and potato onion in different treatments were gently collected from soils and washed with tap water, then washed with autoclaved water. Cleaned roots were completely submerged in a beaker with 200 mL of autoclaved deionized water, and were placed in a plant growth chamber for 6 h at 24°C with light. During the collections, each beaker contained 6 seedlings and covered by tinfoil to avoid contamination and light. Thereafter, the exudates solution were concentrated by freeze-dry and adjusted with autoclaved deionized water to 1 g fresh weight of root per 10 mL exudate solution (1 g FW·10 mL^−1^) (Hao et al., 2010; Li et al., 2013). The root exudates were filtered through a 0.22 μm millipore filters and stored at −20°C up to further investigation.

Poison food technique was used to measure the antifungal activity of the root exudates *in vitro* (Gao et al., 2014). Briefly, 2 mL root exudates of each treatment (OM, OC, TM, TC) were added to Potato Dextrose Agar medium (PDA) (Robb et al., 2009) before it solidified, to yield a total volume of 20 mL per Petri dish (90 × 15 mm). A mycelia disc (5 mm in diameter) was taken out from 10 days-old culture of Vd1 and placed in the center of test Petri dish to observe the growth of mycelia. In order to evaluate the inhibition of the root exudates, the colony diameters were measured using a ruler in three directions on each plate after incubation for 6 days. For determination of spore germination, the spore suspension was diluted to about 1 × 10^3^ spores·mL^−1^ in autoclaved distilled water. Aliquots of 100 μL diluted suspension were spread on the Petri dish. The colonies, which were generated by single germinated spore, respectively, were counted to express the germinated spores after 3 days of incubation. In both measurements 2 mL autoclaved deionized water was used in the control (CK) and the fungus were incubated at 23°C in the dark (Robb et al., 2009). Each treatment had five replicates.

### Analysis of differently expressed genes (DEGs) in tomato roots by RNA-seq

#### Plant material, pathogen inoculation, and sample collection

The procedure used for preparing the tomato seedlings was the same as the one for the collection of root exudates. Briefly, this experiment had two treatments, TM and TC. The roots of tomato plants were collected at 3 DAI. Three samples were prepared for each treatment, respectively, and each sample was pooled from the roots of 5 seedlings. The samples were named TM1, TM2, TM3, and TC1, TC2, TC3, flash frozen in liquid nitrogen, and stored at −80°C until using for the RNA extraction and RNA-seq library preparation.

#### RNA-seq library preparation and sequencing

Total RNA was extracted from different samples (TM1, TM2, TM3, and TC1, TC2, TC3), respectively and treated with DNase I to degrade any possible DNA contamination using RNAprep pure Plant Kit (TIANGEN, China) as described by the manufacturer. RNA was quantified using Agilent 2100 Bioanalyzer (Agilent Technologies, USA), the quality and integrity were assessed by NanoDrop. Quality, quantity and integrity of total RNA from different samples were qualified for RNA-Seq library preparation and sequencing (Additional file 1: Table S1).

RNA-Seq library preparation and sequencing were conducted in BGI Tech (Shenzhen, China). Stepwise below, the mRNA was enriched by using the oligo (dT) magnetic beads, then fragmented into short fragments and mixed with the fragmentation buffer. The first strand of cDNA was synthesized by using random hexamer-primer, and the second strand by addition of Buffer, dNTPs, RNase H and DNA polymerase I. Magnetic beads were used to purify the double strand cDNA. End reparation and 3′- end single nucleotide A (adenine) addition is then performed. At last, sequencing adaptors were ligated to the fragments. The fragments were enriched by PCR amplification. The library products were qualified and quantified by Agilent 2100 Bioanaylzer and ABI StepOnePlus Real-Time PCR System, followed with sequencing by Illumina HiSeq™ 2000.

#### Analysis of illumina sequencing results

Primary sequencing data (called raw reads) produced by Illumina HiSeq™ 2000 were cleaned by discarding reads with adapters and reads in which unknown bases are more than 10%. Low quality reads (sequencing quality is no more than 5) were removed as well. BWA (Li and Durbin, 2009) was used to map clean reads to tomato genome reference, and Bowtie (Langmead et al., 2009) to gene reference using the default parameters, respectively. Genes expression levels in terms of transcripts were quantified by RSEM (RNASeq by Expectation Maximization) and FPKM (Fragment Per Kilobase of exon model per Million mapped reads) method (Li and Dewey, 2011). The FPKM between the biological replications was analyzed by Pearson correlation. In our study, the Pearson coefficient of gene expression in different replications was more than 0.85, indicating the consistency between the replicates (Additional file 2: Figure S1). According to the studies by Tarazonz et al., the Noiseq method was selected to screen differential expressed genes (DEGs) between group TM and TC, with diverge probability (P_NOI_) ≥ 0.8 and the absolute value of fold change ≥ 2 (${\text{log}}_{2}^{\text{Ratio}}\geq 1$) as the threshold value (Tarazona et al., 2011). The expression patter analysis of DEGs is performed with cluster and java Treeview software. WEGO was used to classify GO function (Ye et al., 2006). Kyoto Encyclopedia of Genes and Genomes (KEGG), the major public pathway-related database, is used to perform pathway enrichment analysis of DEGs with Q ≤ 0.05 as significantly enriched threshold (Kanehisa et al., 2008).

#### Verification of RNA-seq results by quantitative real-time PCR (qRT-PCR)

Twelve genes were randomly chosen for verification of RNA-seq results by qRT-PCR. Gene-specific primers were designed using Primer 5.0 software and synthesized by Sangon Biotech Company (Shanghai, China). The genes and primer sequences were listed in Additional file 3: Table S2. The same RNA samples used for RNA-seq library preparation and sequencing were used for the qRT-PCR validation. First-strand cDNA was synthesized with 2 μg of total RNA for reverse transcribing in a 20 μL reaction system using the TIANScript RT Kit. The qRT-PCR reactions were performed on iQ5 Multicolor Real-Time PCR Detection System (BIO-RAD, USA), using RealmasterMix (SYBR Green) (TIANGEN, China) according to the manufacturer's protocol. Each sample was analyzed with three replicates. The mRNA expression levels of the target genes were normalized relative to the expression of tomato *Actin* gene (Løvdal and Lillo, 2009; Yang et al., 2015) and calculated using the 2^−ΔΔCt^ method.

#### Statistical analysis

In each experiment, the treatments were arranged in triplicate. Every experiment was conducted twice independently except RNA-seq analysis. SPSS 16.0 analysis software (SPSS Inc., USA) was used for statistical analysis. Differences between both treatment groups were tested by the independent sample *t*-test at *p* = 0.05 level. Analysis of variance (ANOVA) was performed among different treated groups, and the means of different treatments were compared by Tukey's tests at *p* = 0.05 level. All data were expressed as mean ± standard error.

## Results

### Effect of companion cropping with potato onion on the incidence and disease severity of tomato verticillium wilt

**Figure 1:**
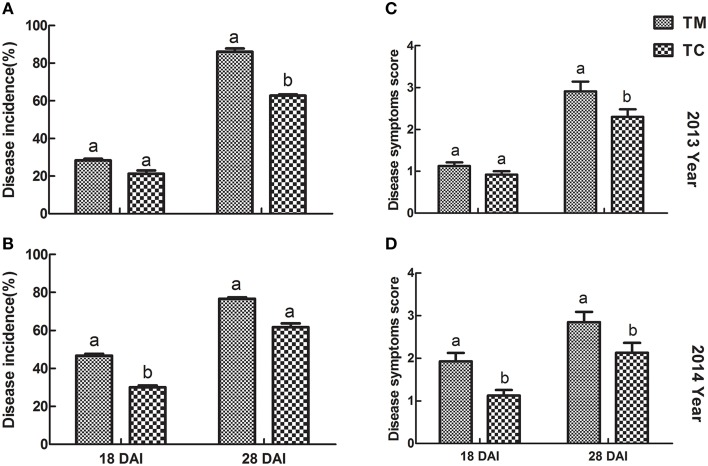
**Disease incidence and disease symptoms score of tomato inoculated with Vd1. (A,B)** Disease incidence of tomato in 2013 and 2014, respectively. **(C,D)** Disease symptoms score in 2013 and 2014, respectively. 18 DAI and 28 DAT indicate 18 and 28 days after infected by Vd1, respectively. Data were the means of three replicates with standards errors shown by vertical bars. The different small letters above the bars represent the significance between two groups of mean values at a lever of *p* = 0.05 according to independent sample *t*-test.

The results regarding to the disease incidence and disease symptoms score of tomato were shown in Figure 1. Compared to TM (tomato monoculture), the disease incidences in TC (tomato companied with potato onion) were decreased by 24.97 and 27.13% in 2013, by 35.58 and 19.83% in 2014 at 18 and 28 DAI, respectively (Figures 1A,B). Similarly, the disease symptoms scores of tomato were significantly declined (*p* ≤ 0.05) in TC compared to TM at all observed stages except 18 DAI in 2013, in which disease symptoms scores was also lower, but not significant (Figures 1C,D).

### Effect of root exudates on Vd1 mycelia growth and spore germination

**Figure 2:**
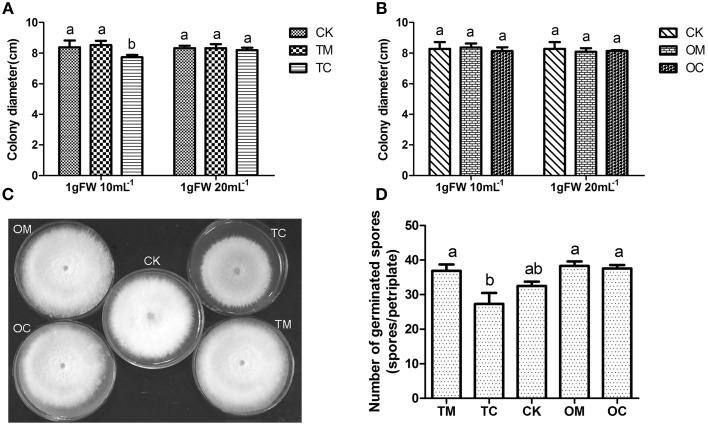
**Effect of root exudates on the mycelia growth and spore germination of Vd1**. **(A)** Effect of root exudates from tomatoes on Vd1 mycelia growth (colony diameter). **(B)** Effect of root exudates from potato onions on Vd1 mycelia growth (colony diameter). **(C)** Effect of root exudates from tomatoes and potato onions on Vd1 mycelia growth (colony diameter) at a concentration of 1 gFW·10 mL^−1^. **(D)** Effect of root exudates from tomatoes and potato onions on Vd1 spore germination at a concentration of 1 g FW·10 mL^−1^. Data are the means of five replicates with standards errors shown by vertical bars. The different small letters above the bars represent among different groups according to Tukey's tests at *p* = 0.05 level.

The whole process for antifungal activities of root exudates was conducted twice independently and the results were shown in Figure 2. In comparison with TM and CK (without root exudates), the root exudates from TC significantly inhibited Vd1 mycelia growth in terms of colony diameter (*p* ≤ 0.05) (Figures 2A,C) with the concentration of 1 g FW·10 mL-1. However, when the concentration was decreased to 1 g FW·20 mL-1, the root exudates from TC had no significant inhibition effect on the Vd1 mycelia growth. In contrast, the root exudates from both OM (potato onion grown alone) and OC (potato grown with tomato) had no significant effect on the mycelia growth at any condition (Figure 2B). Effects of root exudates on Vd1 spore germination were similar to those on Vd1 mycelia growth (Figure 2D).

### Expression profile analysis of tomato root exposed to Vd1 for 3 days by RNA-seq

#### RNA-seq library sequencing and sequencing quality evaluation

Six RNA-seq libraries (TM1, TM2, TM3, and TC1, TC2, TC3) were sequenced and the raw reads were deposited in the NCBI Sequence Read Archive database (Accession SRP057823). After removing the low quality reads, the total number of clean reads in each library ranged from 12.16 to 12.17 million, accounted for 99.78 to 99.87% of total reads (Additional file 4: Figure S2). Base Composition and Quality Distribution of clean data (Additional file 5: Figure S3), Sequencing Saturation Analysis (Additional file 6: Figure S4), as well as Reads Distribution on Gene (Additional file 7: Figure S5) were used for sequence quality assessment. The results showed the sequencing of all the samples had good quality for further analysis.

#### Differential expressed genes (DEGs) analysis and verification of RNA-seq results

**Figure 3:**
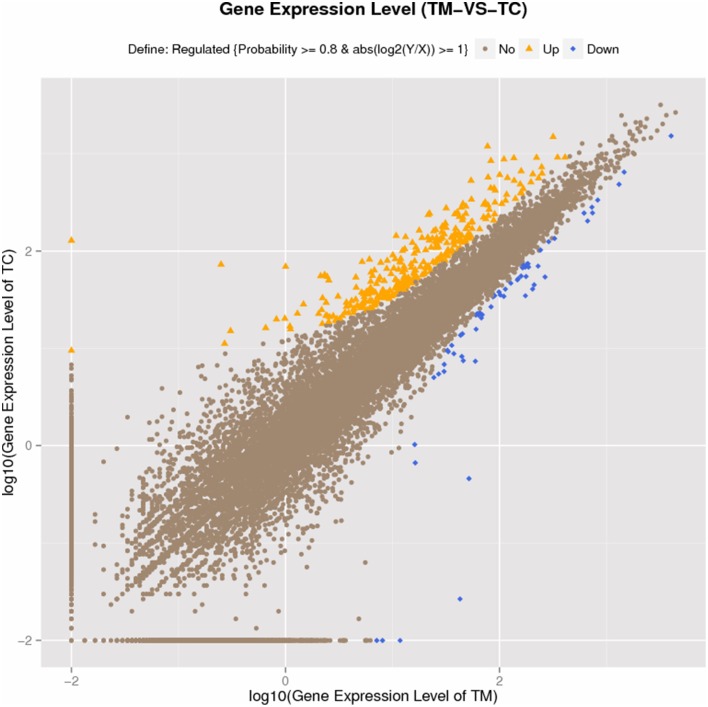
**Gene expression level in TC compared to TM**.

The reads from each sample were aligned to the *Solanum lycopersicum* reference genome, the alignment data and assembly statistics were listed in Table 1. DEGs were screened by Noiseq method, because it had a good performance (Tarazona et al., 2011), with the probability (P_NOI_) = 0.8 as threshold value as described in many reports (Tarazona et al., 2011; Zheng and Moriyama, 2013; Xu et al., 2015b). The results from Noiseq analysis showed there were total 369 DEGs between TM and TC, among which 307 DEGs were up-regulated and 62 DEGs were down-regulated in terms of expression pattern, indicating the up-regulated genes were far more than down-regulated (Figure 3). All DEGs were listed in Additional file 8: Table S3. But we found the log2 values of the DEGs screened by Noiseq method were not very high (Additional file 8: Table S3). To confirm the RNA-seq results, the expression of 12 genes which contain eight up-regulated genes, two down-regulated genes and two genes without variation were quantified by qRT-PCR. The results of the qRT-PCR analysis were consistent to those obtained by RNA-seq analysis except *Aquaporin* gene (Solyc12g044330.1.1), which was no variation between groups in qRT-PCR result, while it was down-regulated in RNA-seq result (Additional file 9: Table S4). This result indicated the Illumina sequencing in our study was of high reliability. Additionally, the Log2 values were not much difference between the two methods (Additional file 9: Table S4), indicating the DEGs screened by Noiseq method was of high reliability, too.

**Table 1:** **Alignment statistics result with reference genome for all samples**.

**Sample**	**Total reads**	**Total base pairs**	**Total mapped reads/**	**Perfect match/match/**	**Mismatch/**	**Unique match/**	**Multi-position match/**	**Total unmapped reads/**
TC1	12173342	596493758	10419039 (85.59%)^*^	8842098 (72.63%)	1576941 (12.95%)	10026087 (82.36%)	392952 (3.23%)	1754303 (14.41%)
TC2	12175372	596593228	10291852 (84.53%)	8683647 (71.32%)	1608205 (13.21%)	9910113 (81.39%)	381739 (3.14%)	1883520 (15.47%)
TC3	12175309	596590141	10280870 (84.44%)	8671568 (71.22%)	1609302 (13.22%)	9888545 (81.22%)	392325 (3.22%)	1894439 (15.56%)
TM1	12172555	596455195	10479406 (86.09%)	9031982 (74.20%)	1447424 (11.89%)	10118908 (83.13%)	360498 (2.96%)	1693149 (13.91%)
TM2	12165484	596108716	10357756 (85.14%)	8719061 (71.67%)	1638695 (13.47%)	9885908 (81.26%)	471848 (3.88%)	1807728 (14.86%)
TM3	12175211	596585339	10470208 (86.00%)	8843302 (72.63%)	1626906 (13.36%)	10065455 (82.67%)	404753 (3.32%)	1705003 (14.00%)

* *Indicates percentages in total reads*.

#### GO analysis for DEGs

**Figure 4:**
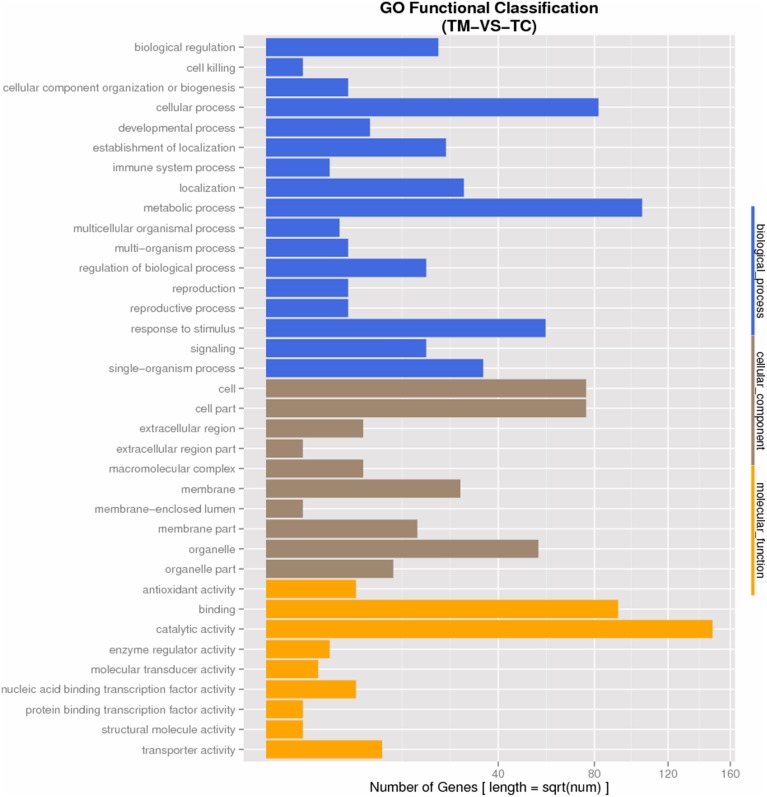
**GO Functional Classification of DEGs**.

GO analysis was used to determine the functions of all DEGs. The DEGs were categorized into three groups as shown in Figure 4. In the cellular component group; cell, cell-part, organelle, membrane-part were the most abundant GO terms induced by TC. Within the biological process categories, metabolic process, cellular process, response to stimulus, biological regulation, localization, regulation of biological process and signaling were dominant terms. The metabolic process, response to stimulus, regulation of biological process and signaling were all involved in the response to the infection of pathogen, indicating the pathogen response related genes were induced in TC. In terms of molecular function, catalytic activity and binging were most GO terms, followed by antioxidant activity, transporter activity, and nucleic acid binding transcription factor activity. All the enriched molecular function categories were involved in the metabolism and transcriptional regulation process in tomato roots exposed to Vd1.

#### Pathway enrichment analysis for DEGs

The 369 DEGs sequences were mapped to the reference canonical pathways in KEGG. A total of 211 DEGs could be annotated in KEGG database and assigned to 65 KEGG pathways. The *metabolic pathway* was the biggest term and contained 70 DEGs occupying 33.18%, followed by *Biosynthesis of secondary metabolites* (54, 25.59%), *Phenylpropanoid biosynthesis* (18, 8.53%), *Zeatin biosynthesis* (18, 8.53%), *Glutathione metabolism* (17, 8.06%), *Plant hormone signal transduction* (17, 8.06%). The summary of significantly enriched KEGG pathways in DEGs was shown in Additional file 10: Table S5, and top 20 enriched KEGG pathways were shown in Table 2.

**Table 2:** **Top 20 enriched KEGG pathways**.

**Pathway**	**DEGs with pathway annotation**	**Pathway ID**
Metabolic pathways	70 (33.18%^*^)	ko01100
Biosynthesis of secondary metabolites	54 (25.59%)	ko01110
Zeatin biosynthesis	18 (8.53%)	ko00908
Phenylpropanoid biosynthesis	18 (8.53%)	ko00940
Glutathione metabolism	17 (8.06%)	ko00480
Plant hormone signal transduction	17 (8.06%)	ko04075
Phenylalanine metabolism	10 (4.74%)	ko00360
Plant-pathogen interaction	10 (4.74%)	ko04626
Flavone and flavonol biosynthesis	9 (4.27%)	ko00944
Flavonoid biosynthesis	9 (4.27%)	ko00941
Cysteine and methionine metabolism	8 (3.79%)	ko00270
Starch and sucrose metabolism	8 (3.79%)	ko00500
Stilbenoid, diarylheptanoid and gingerol biosynthesis	7 (3.32%)	ko00945
Benzoxazinoid biosynthesis	6 (2.84%)	ko00402
Oxidative phosphorylation	6 (2.84%)	ko00190
Cyanoamino acid metabolism	5 (2.37%)	ko00460
Cutin, suberine, and wax biosynthesis	5 (2.37%)	ko00073
Ascorbate and aldarate metabolism	5 (2.37%)	ko00053
Carotenoid biosynthesis	5 (2.37%)	ko00906
ABC transporters	5 (2.37%)	ko02010

* *Indicates the percentage of the total DEGs*.

#### DEGs involved in the functions of disease defense

Transcriptional levels of the genes coding for *Phenylalanine ammonia-lyase* (PAL), Trans-cinnamate 4-monooxygenase, Ferulate-5-hydroxylase, *4-coumarate-CoA Ligase-like protein*, Laccase, and Peroxidase 3 were much higher in TC than the ones in TM (Table 3), especially for Phenylalanine ammonia-lyase gene (Solyc03g071870.1.1) and Laccase gene (Solyc04g072280.2.1), they were 20 and 22 times higher in TC than the ones in TM, respectively. It was notable that all these genes were involved in the biosynthesis of lignin.

**Table 3:** **Expression patterns of DEGs related to biosynthesis of lignin**.

**Gene ID**	**Ratio of FPKM (TC/TM)**	**Probability**	**Description**
Solyc03g071870.1.1	20.49	0.844008772	Phenylalanine ammonia-lyase
Solyc10g011920.1.1	5.99	0.863015339	Phenylalanine ammonia-lyase
Solyc10g011930.1.1	10.15	0.906945967	Phenylalanine ammonia-lyase
Solyc00g282510.1.1	7.30	0.898564424	Phenylalanine ammonia-lyase
Solyc09g007910.2.1	3.38	0.814515824	Phenylalanine ammonia-lyase
Solyc05g047530.2.1	5.34	0.810640148	Trans-cinnamate 4-monooxygenase
Solyc00g247300.2.1	2.97	0.831442762	Ferulate-5-hydroxylase
Solyc06g035960.2.1	6.41	0.873035379	4-coumarate-CoA ligase-like protein
Solyc03g097500.2.1	5.37	0.863885003	Hydroxycinnamoyl CoA shikimate
Solyc03g097500.2.1	8.04	0.886628603	Hydroxycinnamoyl transferase
Solyc04g078660.1.1	3.39	0.822122232	Hydroxycinnamoyl transferase
Solyc01g107080.2.1	22.22	0.897934233	Laccase
Solyc04g072280.2.1	4.18	0.800374334	Laccase
Solyc06g050530.2.1	2.56	0.800078144	Laccase 1a
Solyc04g054690.2.1	2.69	0.810375468	Laccase-13
Solyc06g082240.2.1	0.40	0.818530142	Peroxidase
Solyc06g082420.2.1	7.75	0.864389156	Peroxidase 3
Solyc02g087070.2.1	3.82	0.829369431	Peroxidase family protein

*Probability ≥ 0.8 and absolute value of Ratio ≥ 2 as the threshold to judge the significance of gene expression difference. FPKM represents the expression level*.

Secondly, the transcriptional levels of some proteins, which were related in antifungal activities, were increased significantly, they were listed in Table 4. The expression of gene coding *Wall-associated receptor kinase-like 20* (Solyc01g100090.1.1) was only observed in TC. The transcripts of genes coding *pathogenesis-related protein STH-2-like* (Solycg05g054380.1.1), *Pathogenesis-related protein PR-1* (Solycg006700.1.1), *Kunitz trypsin inhibitor* (Solyc03g098740.1.1), and *Kunitz-type protease inhibitor* (Solyc03g019690.1.1) were higher more than 10 times in TC compared to the ones in TM.

**Table 4:** **Expression patterns of DEGs related to disease defense**.

**Gene ID**	**Ratio of FPKM (TC/TM)**	**Probability**	**Description**
Solyc10g079860.1.1	4.22	0.813205026	Beta-1 3-glucanase
Solyc10g055800.1.1	2.23	0.800084446	Chitinase
Solyc02g082920.2.1	6.96	0.877333283	Endochitinase
Solyc05g054380.1.1	11.08	0.833408956	Pathogenesis-related protein STH-2-like
Solyc08g080670.1.1	2.86	0.826678514	PR5-like protein precursor
Solyc02g065470.1.1	3.77	0.841506913	Pathogenesis-related protein
Solyc00g174340.1.1	2.50	0.817578553	Pathogenesis-related protein 1b
Solyc07g006700.1.1	14.86	0.895180298	Pathogenesis-related protein PR-1
Solyc10g081980.1.1	2.53	0.804735257	Harpin-induced protein-like
Solyc02g036480.1.1	6.59	0.881316091	Harpin-induced protein-like
Solyc03g098730.1.1	4.96	0.826401231	Kunitz trypsin inhibitor ST1-like
Solyc03g098740.1.1	10.90	0.902559837	Kunitz trypsin inhibitor
Solyc03g019690.1.1	12.28	0.853587679	Kunitz-type protease inhibitor
Solyc03g020010.1.1	5.01	0.870766691	Kunitz-type trypsin inhibitor alpha chain
Solyc09g082300.2.1	5.79	0.819311579	Non-specific lipid-transfer protein
Solyc09g065440.2.1	4.88	0.837675351	Non-specific lipid-transfer protein
Solyc09g065430.2.1	3.31	0.845225041	Non-specific lipid-transfer protein
Solyc01g005990.2.1	4.51	0.854652702	Non-specific lipid-transfer protein
Solyc08g067500.1.1	0.37	0.833383749	Non-specific lipid-transfer protein
Solyc03g005210.2.1	2.98	0.815738395	Non-specific lipid-transfer protein
Solyc01g103060.2.1	3.32	0.822323893	Non-specific lipid-transfer protein
Solyc09g082270.2.1	3.58	0.858074643	Non-specific lipid-transfer protein
Solyc01g081600.2.1	3.03	0.831764157	Non-specific lipid-transfer protein
Solyc10g075100.1.1	8.67	0.899156804	Non-specific lipid-transfer protein
Solyc08g062220.2.1	5.94	0.843693677	UDP-glucose glucosyltransferase
Solyc08g006330.2.1	2.93	0.800727241	UDP-glucose salicylic acid glucosyltransferase
Solyc11g007390.1.1	9.35	0.878417212	UDP-glucosyltransferase
Solyc11g007490.1.1	5.85	0.875783013	UDP-glucosyltransferase
Solyc01g095620.2.1	2.92	0.836320442	UDP-glucosyltransferase
Solyc11g007500.1.1	4.94	0.884372519	UDP-glucosyltransferase
Solyc07g043150.1.1	3.90	0.859505174	UDP-glucosyltransferase
Solyc02g085660.1.1	8.89	0.882551266	UDP-glucosyltransferase
Solyc12g042600.1.1	5.92	0.888733442	UDP-glucosyltransferase family 1 protein
Solyc01g107780.2.1	3.60	0.858534679	UDP-glucosyltransferase family 1 protein
Solyc01g107820.2.1	4.15	0.877043395	UDP-glucosyltransferase family 1 protein
Solyc10g079930.1.1	3.58	0.853965793	UDP-glucosyltransferase HvUGT5876
Solyc07g006800.1.1	4.62	0.869689064	UDP-glucosyltransferase HvUGT5876
Solyc12g057080.1.1	3.82	0.831505779	UDP-glucuronosyltransferase
Solyc12g057060.1.1	3.01	0.848394903	UDP-glucuronosyltransferase
Solyc03g078490.2.1	6.36	0.845697685	UDP-glucuronosyltransferase
Solyc03g071850.1.1	3.47	0.854173756	UDP-glucuronosyltransferase 1-6
Solyc12g009930.1.1	2.69	0.8174021	UDP-glucuronosyltransferase 1-6
Solyc10g085890.1.1	4.03	0.805838091	UDP-glycosyltransferase 73C3-like
Solyc01g100090.1.1	127.98/0.01^*^	0.988511614	Wall-associated receptor kinase-like 20
Solyc01g080010.2.1	4.91	0.861257105	Xylanase inhibitor (Fragment)

*Probability ≥ 0.8 and the absolute value of Ratio (TC/TM) ≥ 2 as the threshold to judge the significance of gene expression difference. FPKM represents the expression level*.

* *Indicates the gene was expressed only in TC (when the value of either sample FPKM was zero, 0.01 was used to instead of 0 to calculate the fold change)*.

Finally, the expressions of genes related to hormone metabolism and signaling pathways were increased by the companion cropping with potato onion (Table 5). The expressions of genes coding *1-aminocyclopropane-1-carboxylate oxidase* (Solycg026650.2.1 and Solyc01g095080.2.1), and *Phenylalanine ammonia-lyase* (Solyc03g071870.1.1 and and Solyc10g011930.1.1) were higher more than 10 times in TC compared to the ones in TM.

**Table 5:** **Expression patterns of DEGs related to hormone metabolism and transcription factors**.

**GeneID**	**Ratio of FPKM (TC/TM)**	**Probability**	**Gene description**
**HORMONE METABOLISM AND SIGNALING-RELATED GENE EXPRESSION**			
Solycg049530.2.1	5.60	0.832236801	1-aminocyclopropane-1-carboxylate oxidase
Solycg026650.2.1	15.26	0.819387202	1-aminocyclopropane-1-carboxylate oxidase
Solyc12g006380.1.1	6.18	0.891739454	1-aminocyclopropane-1-carboxylate oxidase-like protein
Solyc09g089580.2.1	5.88	0.826911685	1-aminocyclopropane-1-carboxylate oxidase-like protein
Solyc04g009860.2.1	3.05	0.822418422	1-aminocyclopropane-1-carboxylate oxidase-like protein
Solyc01g095080.2.1	13.46	0.894367351	1-aminocyclopropane-1-carboxylate synthase
Solyc06g053710.2.1	4.65	0.831587704	Ethylene receptor
Solyc08g014000.2.1	0.42	0.804476878	Lipoxygenase
Solyc08g029000.2.1	3.90	0.824378316	Lipoxygenase
Solyc03g122340.2.1	4.12	0.810173807	Lipoxygenase
Solyc12g013620.1.1	4.87	0.874434403	NAC domain protein IPR003441(jasmonic acid 2)
Solyc03g071870.1.1	20.49	0.844008772	Phenylalanine ammonia-lyase
Solyc10g011920.1.1	5.99	0.863015339	Phenylalanine ammonia-lyase
Solyc10g011930.1.1	10.15	0.906945967	Phenylalanine ammonia-lyase
Solyc00g282510.1.1	7.30	0.898564424	Phenylalanine ammonia-lyase
Solyc09g007910.2.1	3.38	0.814515824	Phenylalanine ammonia-lyase
Solyc08g062220.2.1	5.94	0.843693677	UDP-glucose salicylic acid glucosyltransferase
Solyc08g006330.2.1	2.93	0.800727241	UDP-glucose salicylic acid glucosyltransferase
Solyc11g007490.1.1	5.85	0.875783013	UDP-glucose salicylic acid glucosyltransferase
Solyc11g007500.1.1	4.94	0.884372519	UDP-glucose salicylic acid glucosyltransferase
Solyc03g078490.2.1	6.36	0.845697685	UDP-glucose salicylic acid glucosyltransferase
**TRANSCRIPTION FACTORS**			
Solyc09g005610.2.1	9.00	0.886849170	BZIP transcription factor TGA2-like
Solyc02g080890.2.1	3.09	0.829923999	Transcription factor WRKY31 isoform X1)
Solyc02g094270.1.1	4.05	0.817843234	WRKY transcription factor 45
Solyc06g066370.2.1	2.68	0.817320175	WRKY transcription factor 1
Solycg05g015850.2.1	6.76	0.846970671	WRKY transcription factor-b
Solyc09g014990.2.1	3.13	0.803871895	WRKY-like transcription factor 26
Solyc09g089930.1.1	4.32	0.816948362	Ethylene responsive transcription factor 1a
Solyc12g056590.1.1	3.80	0.816948362	Ethylene responsive transcription factor 2a
Solyc04g071770.2.1	4.32	0.833591712	Ethylene responsive transcription factor 2a
Solyc09g075420.2.1	4.61	0.864439571	Ethylene responsive transcription factor 2b
Solyc09g091950.1.1	0.34	0.875776711	Ethylene-responsive transcription factor 1
Solyc02g077370.1.1	3.75	0.810993055	Ethylene-responsive transcription factor 2
Solyc01g104740.2.1	6.49	0.887132756	Ethylene-responsive transcriptional coactivator
Solyc10g008700.1.1	6.47	0.818990182	MYB transcription factor
Solyc09g090790.2.1	4.79	0.848502036	MYB transcription factor
Solyc12g099130.1.1	6.62	0.807974439	MYB transcription factor
Solyc02g089190.1.1	8.78	0.822746121	MYB transcription factor
Solyc03g093890.2.1	4.18	0.826130248	Myb-related transcription factor
Solyc02g089190.1.1	3.13	0.817843234	Susceptibility homeodomain transcription factor
Solyc03g093890.2.1	4.00	0.830094151	Sigma factor binding protein 1
Solycg05g054650.1.1	4.09	0.821567664	Zinc finger transcription factor ZFP19
Solyc01g096510.2.1	2.67	0.817206741	Zinc finger AN1 domain-containing stress-associated protein
Solyc02g087210.2.1	4.30	0.876198939	Zinc finger family protein C2H2-type)
Solycg05g054650.1.1	4.09	0.821567664	Zinc finger transcription factor ZFP19

*Probability ≥ 0.8 and the absolute value of Ratio ≥ 2 as the threshold to judge the significance of gene expression difference. FPKM represents the expression level*.

## Discussion

### Companion cropping with potato onion alleviated the disease development of tomato verticillium wilt

In this study, the results showed that companion cropping with potato onion could alleviate disease development of tomato Verticillium wilt based on the disease incidence and disease symptoms score when tomato was companied with potato onion (Figure 1). These were consistent with the previous studies (Ren et al., 2008; Gao et al., 2014; Yang et al., 2014), which demonstrated intercropping could suppress soil-borne disease. However, the field study is needed further to confirm the phenomenon, because some other factors may affect the results. As Gao et al., and Yang et al., reported, the horizontal distance between two plant species could be involved in the efficiency of disease controlling (Gao et al., 2014; Yang et al., 2014).

In field, some other factors could affect the incidence and disease severity of soil-borne disease, such as the alteration of microenvironment and form of “root wall” (Gómez-Rodrıguez et al., 2003; Gao et al., 2014), or/and direct inhibition of pathogens with root exudates from intercropped plants (Ren et al., 2008; Hao et al., 2010; Gao et al., 2014; Yang et al., 2014). These factors could decrease the number of pathogens which attack plants. But in our studies, each tomato seedling was inoculated artificially with the same pathogen, therefore, the effects of these other factors could be negligible, indicating the efficiency of disease controlling in our study was mainly depended on the disease resistance of tomato.

### Companion cropping with potato onion could induce the antifungal activity of tomato root exudates on the inhibition of *V. dahliae* growth

Previous studies indicated that the antifungal activity of root exudates may inhibit the growth of soil-borne pathogens in intercropping system. However, all of the researches were focused on the effects of the root exudates from companion plants on the pathogens (Ren et al., 2008; Hao et al., 2010; Gao et al., 2014). Interestingly, in this study, the root exudates from the companion plant (potato onion) had no effects on *V. dahliae*, instead the root exudates from host plant (tomato) did (Figure 2). The results obtained from our study suggest that the tomato was induced to produce an antifungal activity to defense the pathogens when it was companied with potato onion. This was similar to the study reported by Gao et al., who demonstrated that the root exudates of maize/soybean intercropped plants inhibited the growth of *Cylindrocladium parasiticum* (Gao et al., 2014). However, in their study, which plant generated the antifungal substance remained unknown. In our study we demonstrated that the antifungal activity of root exudates came from tomato, which was the host plant instead of the companion plant. To our knowledge, our result is the first report to demonstrate that intercropping could induce the antifungal activity of root exudates from the host plant.

Interestingly, our results posed a new scientific problem. That was what induced the antifungal activity of root exudates from host plant (tomato)? As Hage-Ahmed et al., have demonstrated the root exudates from the tomatoes which were inoculated with arbuscular mycorrhizal fungi (AMF) and *Fusarium oxysporum* f.sp. *lycopersici (Fol) could* inhibit the spore germination *of Fol*, rather than those inoculated with AMF or *Fol* individually (Hage-Ahmed et al., 2013). It suggested that the interactions of tomato- AMF- *Fol* induced the generation of antifungal substances for the defense to pathogens. In our study, it was the root exudates from tomato which was companied with potato onion, together with the infection by *V. dahliae*, was induced to generate the antifungal activity against *V. dahliae*. However, it was unknown that this antifungal activity of root exudates was generated before or after the infection by *V. dahliae?* In other words, the problem on which this antifungal activity was induced by the interactions of plant (tomato)-plant (potato onion) or plant (tomato)-plant (potato onion)-pathogen (*V. dahliae)* deserved further investigation.

### Companion cropping with potato onion could enhance the expression of genes involved in disease resistance of tomato against *V. dahliae*

The generation of antifungal activity in the root exudates of tomato increased disease resistance of tomato. To explore how the disease resistance of tomato was enhanced in transcriptional level, RNA-Seq was employed. The results showed that the expressions of many genes were changed in the tomato companioned with potato onion. By further analysis of KEGG, it was found that the most affected genes were the genes that were involved in *metabolic pathway* and *biosynthesis of secondary metabolites* in the tomato (Table 2), which suggest that the metabolism was enhanced in the root of tomato grown with potato onion. It was deduced that the antifungal activity was produced by secondary metabolite(s), and the metabolite(s) was generated by the genes, which were involved in the metabolism pathway.

The DEGs analysis showed that the expressions of genes involved in disease defense were increased in the roots of tomato companied with potato onion. Without a doubt, the increases on these gene expressions were beneficial to the disease resistance of tomato. Lignin was considered to function as a physical barrier against infection of pathogens (Underwood, 2012). The expression levels of genes involved in biosynthesis of lignin were higher in roots of tomato companied with potato onion compared to that in tomato grown alone (Table 3). This was consistent with previous studies in the watermelon challenged with *Fusarium oxysporum* f.sp. niveum and in the banana infected with *Fusariu m oxysporum* f.sp. *cubense* Tropical Race 4 (Lü et al., 2011; Bai et al., 2013).

Pathogenesis-related proteins (PRs) are important inducible defense-related proteins upon infection with various pathogens (van Loon et al., 2006). In our study the expressions of genes coding *PR5, PR1, PR6, PR1b, Endochitinase*, and *Beta-1,3-glucanase*, which are all PRs, were significantly increased in the tomato companied with potato onion compared to the one in the tomato grown alone (Table 4). This was similar to the previous studies, which showed that intercropping could induce the expression of PR genes in a high level (Schmid et al., 2013; Gao et al., 2014). There were some other defense-related proteins except PRs in plant defense response system, such as *Kunitz trypsin inhibitor* (Huang et al., 2010), *Non-specific lipid-transfer protein* (Wang et al., 2004), *UDP-glucose salicylic acid glucosyltransferase* (Sepúlveda-Jiménez et al., 2005), *Wall-associated receptor kinase* (Li et al., 2009), *Xylanase inhibitor* (Sansen et al., 2004), and so on. The genes coding for the proteins were expressed higher in root of tomato grown with potato onion than that in the tomato grown alone (Table 4).

Plant hormones can regulate the expressions of defense-related genes, especially SA, JA and Ethylene (Wang et al., 2002; Denance et al., 2013). In our study, the expression of genes involved in biosynthesis of Ethylene and response to Ethylene signaling were all up-regulated in tomato companied with potato onion compared to tomato grown alone (Table 5). In terms of SA, the expression of genes involved in SA signaling, such as *TGA transcription factor 2* gene, *WRKY transcription factor* gene and *PR-1 protein gene* were all increased in tomato companied with potato onion (Table 5). These results suggested that Ethylene and SA played important roles in the defense response of tomato against *V. dahliae*.

The up-regulated expression of genes related to disease response suggested the disease resistance of the tomato companied with potato onion was enhanced compared to that grown alone, in transcriptional level. Together with the decrease of disease development of tomato Verticillium wilt (in morphological level), and induced antifungal activity of root exudates from tomato companied with potato onion (in physiological level), the three independent studies demonstrated the disease resistance of tomato was enhanced against *V. dahliae*. This may be a potential mechanism for the management of soil-borne plant diseases in the intercropping system. However, what induced the enhancement of disease resistance of tomato in intercropping was unclear. As Schmid et al., have demonstrated interspecific interactions between *Arabidopsis thaliana* (plant) and *Hieracium pilosella* (plant) could highly induce the expression of PR genes (Schmid et al., 2013). Wang et al. observed the changes of activity levels of antioxidases and content of malondialdehyde which were related to stress resistance in the eggplant intercropped with garlic (Wang et al., 2015). These suggested interspecific interactions of plant-plant could induce the resistance response of intercropped plants. But Hage-Ahmed et al., have demonstrated the antifungal activity of root exudates from the tomato was induced by the interactions of plant- AMF- pathogen (Hage-Ahmed et al., 2013). So in our studies, the problem on which the enhancement of disease resistance in tomato root in the intercropping was induced by the interactions of plant (tomato)—plant (potato onion), or by the interactions of plant (tomato)—plant (potato onion)—pathogen (*V. dahliae)* deserved further investigation.

## Conclusion

The results obtained from this study indicated that the companion cropping with potato onion could decrease the incidence of tomato Verticillium wilt and alleviate the disease severity levels. The further study showed the tomato companied with potato onion was induced to produce antifungal activity for the inhibition of *V. dahliae* growth via root exudates under the condition of infection with *V. dahliae*. Meanwhile, the expressions of genes related to disease resistance were higher in tomato companied with potato onion compared to those in tomato grown alone. Based on the results, we proposed that companion cropping with potato onion enhance the disease resistance of tomato against *V. dahliae*, by inducing the antifungal activity of root exudates from tomato, and by the up-regulated expression of genes involved in defense response to pathogen. This may be a potential mechanism for the management of tomato Verticillium wilt in tomato/potato onion companion cropping.

### Conflict of interest statement

The authors declare that the research was conducted in the absence of any commercial or financial relationships that could be construed as a potential conflict of interest.
