# Supplementary material for: Companion cropping with potato onion enhances the disease resistance of tomato against Verticillium dahliae
Source: Front Plant Sci. 2015 Sep 11;6:726. doi: 10.3389/fpls.2015.00726 (PMC4566073; doi:10.3389/fpls.2015.00726)
Supplement: Supplementary file 4 [file Table4.DOC]

**Companion cropping with potato onion enhances the disease resistance of tomato against *Verticillium dahliae***

**Xuepeng Fu, Xia Wu,Xingang Zhou,Shouwei Liu,Yanhui Shen,Fengzhi Wu**

**Additional file 9, Table S4. qRT-PCR confirmation of select DEGs identified by RNA-seq.**

|  |  | Expression | **log_2_fold-change** | |
| --- | --- | --- | --- | --- |
| **GeneID** | **Genes Description** | variation | **qRT-PCR** | **RNA-seq** |
| Solyc01g095080.2.1 | 1-aminocyclopropane-1-carboxylate synthase | UP | 1.79 | 3.75 |
| Solyc06g053710.2.1 | Ethylene receptor | UP | 1.09 | 2.22 |
| Solyc02g077370.1.1 | Ethylene-responsive transcription factor 2 | UP | 2.55 | 1.91 |
| Solyc10g079860.1.1 | Beta-1 3-glucanase | UP | 1.79 | 2.08 |
| Solyc07g006700.1.1 | Pathogenesis-related protein | UP | 1.69 | 3.89 |
| Solyc02g065470.1.1 | Pathogenesis-related protein | UP | 1.12 | 1.91 |
| Solyc08g074680.2.1 | polyphenol oxidase | No variation | 0.33 | 0.74 |
| Solyc06g060970.1.1 | Expansin-like protein | UP | 6.00 | 6.1 |
| Solyc09g082550.2.1 | High affinity sulfate transporter 2 | UP | 4.32 | 4.14 |
| Solyc06g049080.2.1 | Superoxide dismutase | No variation | 0.55 | -0.13 |
| Solyc12g044330.1.1 | Aquaporin | down | -0.76 | -1.21 |
| Solyc11g069760.1.1 | High affinity nitrate transporter protein | down | -1.55 | -2.62 |

Note:Values are shown as log_2_ratio.The data of qRT-PCR are means of three independent biological with three technical replicates. TM was served as control group and TC as treatment group using the 2^−ΔΔCt^ method. The RNA-seq results are absolute quantification while qRT-PCR results are relative quantification to reference gene.
