# Supplementary figures and images for: Companion cropping with potato onion enhances the disease resistance of tomato against Verticillium dahliae
Source: Front Plant Sci. 2015 Sep 11;6:726. doi: 10.3389/fpls.2015.00726 (PMC4566073; doi:10.3389/fpls.2015.00726)

Additional file 2: Fig. S1 The Pearson coefficient of gene expression in different replicates


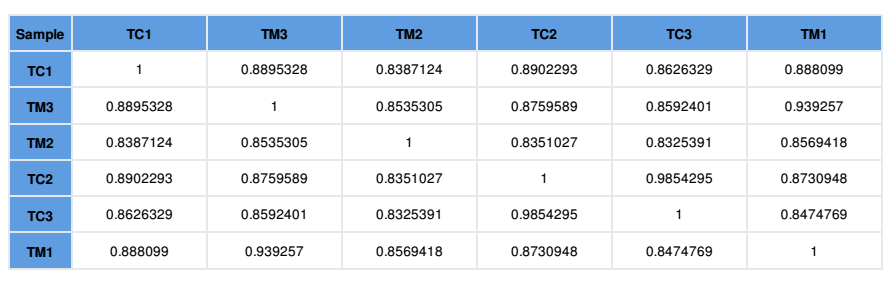

Supplement: Supplementary file 6 [file DataSheet1.DOC]
