# Supplementary material for: Companion cropping with potato onion enhances the disease resistance of tomato against Verticillium dahliae
Source: Front Plant Sci. 2015 Sep 11;6:726. doi: 10.3389/fpls.2015.00726 (PMC4566073; doi:10.3389/fpls.2015.00726)

**Companion cropping with potato onion enhances the disease resistance of tomato against *Verticillium dahliae***

**Xuepeng Fu, Xia Wu,Xingang Zhou,Shouwei Liu,Yanhui Shen,Fengzhi Wu**

**Additional file 3, Fig.S2 Classification of raw reads**


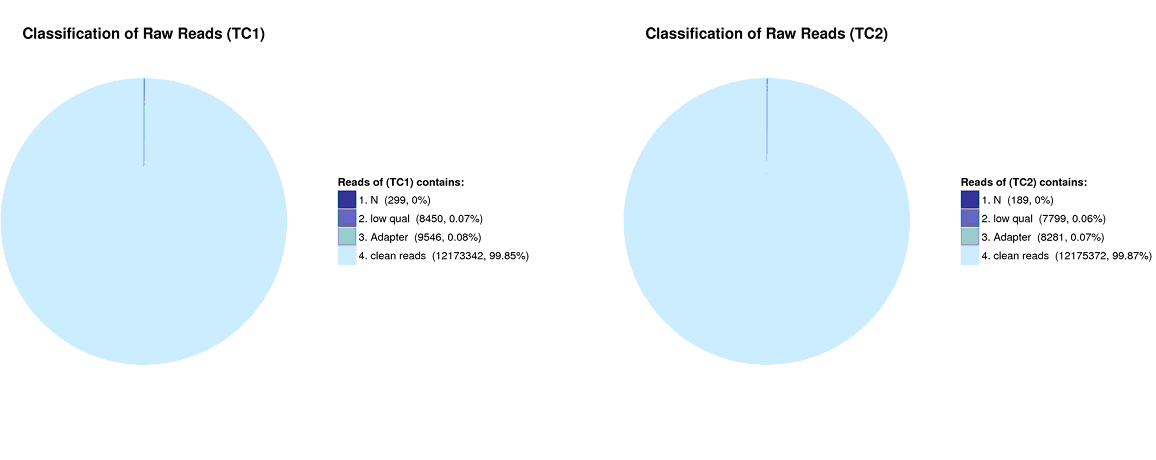

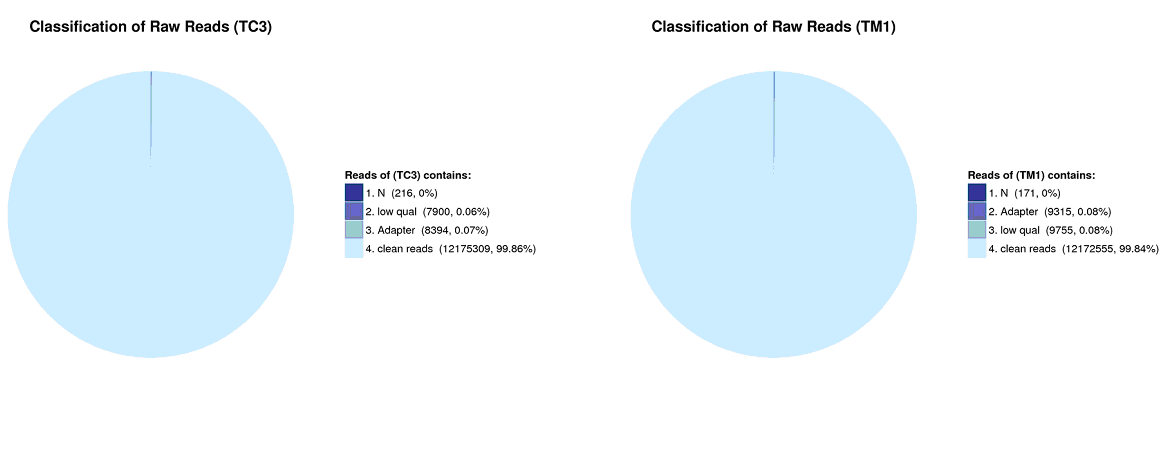

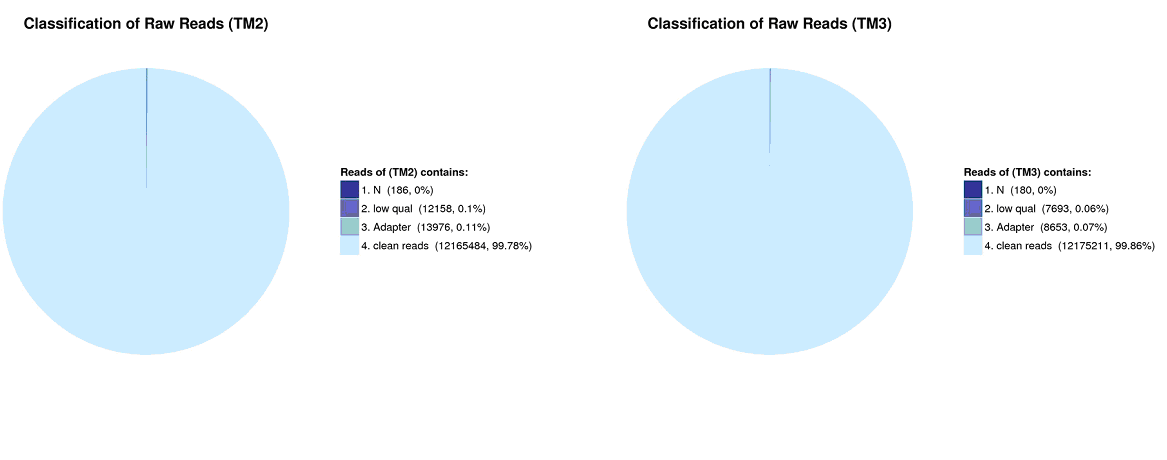

Supplement: Supplementary file 7 [file DataSheet2.DOC]
